# An Adaptive Fault-Tolerant Event Detection Scheme for Wireless Sensor Networks

**DOI:** 10.3390/s100302332

**Published:** 2010-03-19

**Authors:** Sung-Jib Yim, Yoon-Hwa Choi

**Affiliations:** Department of Computer Engineering, Hongik University, 72-1 Sangsu-Dong, Mapo-Gu, Seoul, Korea; E-Mail: yimsungjib@gmail.com

**Keywords:** sensor networks, fault tolerance, event detection, adaptive

## Abstract

In this paper, we present an adaptive fault-tolerant event detection scheme for wireless sensor networks. Each sensor node detects an event locally in a distributed manner by using the sensor readings of its neighboring nodes. Confidence levels of sensor nodes are used to dynamically adjust the threshold for decision making, resulting in consistent performance even with increasing number of faulty nodes. In addition, the scheme employs a moving average filter to tolerate most transient faults in sensor readings, reducing the effective fault probability. Only three bits of data are exchanged to reduce the communication overhead in detecting events. Simulation results show that event detection accuracy and false alarm rate are kept very high and low, respectively, even in the case where 50% of the sensor nodes are faulty.

## 1. Introduction

Wireless sensor networks often consist of a large number of small sensor nodes that cooperate to monitor real-world events and enable applications such as target tracking, military tactical surveillance, and emergency health care [1]. The detection and reporting of the occurrence of an interesting event is one of the important tasks of sensor networks. Due to limitations in available resources, such as power, memory and computing capability, sensor nodes deployed in a harsh environment, operating in an unattended mode, are prone to failure. Faulty nodes might issue an alarm even though they are not in an event region. They degrade the network reliability, unless some provisions are made to tolerate them.

Several distributed schemes for detecting events in the presence of faulty sensor nodes have been proposed in [2–5]. Krishnamachari and Iyengar [2] have mathematically proven that the majority voting is an optimal decision for the given model to detect events and correct faults. A single binary variable is used to represent a local event detection, resulting in low communication cost. Their simulation results show that 85∼95% of faults can be reduced when fault rate is about 10%. Luo *et al.* [3] proposed a fault-tolerant energy-efficient event detection paradigm for wireless sensor networks. For a given detection error bound, minimum neighbors are selected to minimize the communication volume. Both Bayesian and Neyman-Pearson detection methods are presented. A localized event boundary detection scheme, exploiting the notion that readings from the event region and the normal region have different means but the same standard deviation due to noise, has been proposed in [4]. Actual sensor readings, encoded in 32 bits each, are transmitted and used in making a decision. The corresponding estimation may be more precise at the cost of increased communication overhead. Jin *et al.* [5] have employed a variable length event coding mechanism in event and event boundary detection to balance the communication cost and the estimation quality. Sensor nodes near the event boundary send the original sensor readings of 32 bits (with a 1-bit flag), whereas all others nodes use only two bits of message, instead.

In [6], a fault-tolerant event boundary detection algorithm using a clustering technique based on maximum spanning trees is presented. Difference in sensor readings between any two sensor nodes is represented as the distance between them. Using the distances sensor nodes are classified into two clusters. With some additional computation on the clusters, event boundary nodes are determined.

Most of the proposed event detection schemes based on a statistical model of noise may work effectively for a relatively low fault probability. As the fault probability increases, however, their performance degrades considerably. Moreover, the actual performance might differ significantly from the estimated one if faults behave differently from the model.

In this paper, we present a distributed adaptive fault-tolerant event detection scheme for wireless sensor networks. It achieves high performance for a wide range of fault probabilities by employing a filter for tolerating transient faults and by dynamically adjusting the threshold for event detection depending on the fault status of sensor nodes. Confidence levels are used to manage the status of sensor nodes. Sensor nodes with a permanent fault (or behaving incorrectly for an extended period of time) are isolated from the network and reinstated later if some required conditions on confidence levels are met. Due to the adaptability of the proposed scheme both high event detection accuracy and low false alarm rate can be maintained even with increasing number of faults.

The remainder of the paper is organized as follows. In Section 2, the system model and fault model are briefly described. Section 3 presents our adaptive event detection scheme employing a dynamic threshold selection. Filtering transient faults is also proposed to reduce the effective fault probability of sensor nodes. Simulation results are shown in Section 4. Conclusions are made in Section 5.

## 2. System Model and Fault Model

As the system model we assume that sensor nodes are randomly deployed in the target area and all sensor nodes have the same transmission range *r*. Each sensor node receives the sensor readings of neighboring nodes and makes a decision on an event locally in a distributed manner. We define the average node degree *d* to represent the connectivity of the network. For convenience an event region is a circle with radius *l*. The proposed adaptive scheme, however, is expected to perform well even with different event region shapes. Each sensor node is assumed to know the range of normal sensor readings, and thus can make a decision on its own whether the sensed data lies in the range of normal readings or not and report a 1(abnormal) or 0(normal) accordingly. Apparently a faulty sensor or an event may produce abnormal data, and thus they are indistinguishable based on the readings of a single sensor node. All the sensor readings are assumed to be binary, without loss of generality. In the case of arbitrary values, comparison diagnosis presented in [7, 8] may be used instead.

Three different types of faults in sensor readings, depending on their temporal behavior, are considered in this paper: permanent, transient, and intermittent [9–11]. In the case of a permanent fault, we assume that it causes an incorrect reading, either 1 or 0, consistently, with the same probability of 0.5, irrespective of the region it is in. Transient faults are assumed to be independent both spatially and temporally. A special type of intermittent fault which generates erroneous data periodically is also taken into account to estimate the adaptability of the proposed scheme. Although we focus on faulty sensors in this paper, the proposed scheme can possibly be extended to cover faulty communications with some degradation in performance by modeling faults in communication as sensor faults in the associated sensor nodes.

Sensor networks are assumed to conduct fault detection periodically to manage fault status of sensor nodes. The period, however, is expected to be long enough to reduce the overhead incurred. Nevertheless the event detection performance can be maintained extremely high as long as most of the faulty sensors nodes are identified and isolated.

## 3. Adaptive Event Detection Scheme

In this section, we first describe the confidence levels of sensor nodes to be used in the proposed event detection scheme. We then present our adaptive event detection scheme using the confidence levels defined. Some erroneous readings due to transient faults will be corrected by employing a moving average filter to further enhance event detection performance. For convenience we list the notation to be used in this paper.

**Table:** Notation

*v_i_*	sensor node
$x_{i}^{k}$	sensor reading at node *v_i_* at time *k*
$y_{i}^{k}$	filtered output of the input $x_{i}^{k}$ (to tolerate most transient faults)
*R_i_*	threshold test result at *v_i_* based on *x_i_* and *x′_j_s* (*i.e.*, neighbors’)
*H_i_*	threshold test result at *v_i_* based on *y_i_* and *y′_j_s*
*D_i_*	final decision on an event at *v_i_*
*F_i_*	fault status of *v_i_* (good, faulty)
*F_ij_*	fault status of *v_j_* from the viewpoint of *v_i_* (good, faulty)
*d_i_*	node degree of *v_i_*
$d_{i}^{k}$	effective node degree of *v_i_* at time *k* (*i.e.*, number of neighboring nodes with *F_ij_* = 0
*l*	radius of an event region
*r*	transmission range
*d*	average node degree of a sensor network (*i.e.*, $d=\frac{\sum d_{i}}{N}$)
*d^k^*	average effective node degree of a sensor network at time *k* (*i.e.*, $d^{k}=\frac{\sum d_{i}^{k}}{N}$)
*M*	window size for tolerating transient faults
*δ*	threshold for filtering transient faults
*c_i_*	self confidence level of *v_i_*
*w_ij_*	confidence level of *v_j_* from the viewpoint of *v_i_*
*p_p_*	permanent fault probability
*p_t_*	transient fault probability
*θ*	threshold for event detection

### 3.1. Confidence Levels

In order to describe confidence levels of a sensor node and its neighbors a sensor network is modeled here as a weighted directed graph, *G*(*V;E*), where *V* represents the set of sensor nodes and *E* represents the set of edges connecting sensor nodes. Two nodes *v_i_* and *v_j_* are said to be connected if the distance between them dist(*v_i_, v_j_*) is less than or equal to *r* (transmission range). Each node *v_i_* is assigned a self-confidence level *c_i_*. Each edge *e_ij_* is also assigned a weight *w_ij_*, indicating the confidence level of *v_j_* from the viewpoint of *v_i_*. The confidence levels will be used to isolate potentially faulty sensor nodes from the rest of the network. They are also used to reinstate an isolated node if the confidence levels associated with it satisfy the required conditions to be addressed shortly. We use *c_min_* and *c_max_* to denote the range of the confidence level *c_i_*. Also *w_min_* and *w_max_* will be used to indicate the range of *w_ij_*.

**Figure 1.**
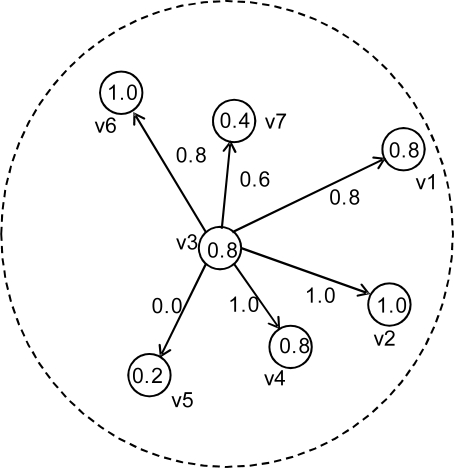
An illustration of confidence levels.

An illustration is given in Figure 1, where six nodes are neighbors of the node *v*_3_ (*i.e.*, six nodes are located within the communication range of *v*_3_) and confidence levels *c_i_* and *w_ij_* are assumed to be in the range of 0 to 1. In the figure, from the viewpoint of node *v*_3_, *v*_2_ and *v*_4_ are nodes with the highest confidence while *v*_5_ is a node with the lowest confidence. Among the six neighboring nodes of *v*_3_, *v*_5_ is the most likely to be faulty, and will be ignored from *v*_3_ if *w_min_* = 0.

The confidence levels will be updated each time a fault detection or event detection is performed. All the *c_i_* and *w_ij_* are initialized to 1 (*i.e.*, *c_max_* and *w_max_*). They are increased or decreased by *α* (0 *< α <* 1) when the required conditions to be explained later are met.

### 3.2. Filtering Transient Faults

Event detection performance will degrade as the fault probability *p* increases. Hence reducing the effective *p* is desirable to make an event detection scheme robust to faults occurring in sensor networks. In order to do that, we use the confidence levels defined above to isolate faulty nodes and employ a modified moving average filter, to be discussed here, to correct some erroneous sensor readings due to transient faults.

Let $x_{i}^{k}$ represent sensor reading at node *v_i_* at time *k*. Then the filter we employ takes an average of the last *M* readings, $x_{i}^{n}$, $x_{i}^{n-1},\ldots ,$ and $x_{i}^{n-M+1}$, and sets the output $y_{i}^{n}$ to 1 if it passes a given threshold *δ*. Hence the output $y_{i}^{n}$ (*i.e.*, filtered output at node *v_i_*) can be expressed as follows:

(1) $$y_{i}^{n}=\{\begin{array}{ll} 1 & \sum_{j=n-M+1}^{n}x_{i}^{j}\geq M\delta ,\\ 0 & \text{otherwise}. \end{array}$$

Parameters, *M* (*i.e.*, window size) and *δ* (threshold) need to be properly chosen, depending on applications, for the best performance. They can be dynamically adjusted to enhance adaptability. As long as most of erroneous readings due to transient faults can be corrected, however, a high event detection performance can be obtained as will be shown in the simulation results in Section 4. Due to the fact that an event may cause abnormal sensor readings for an extended period of time, most transient faults can be filtered unless they occur repeatedly within the window. Although the types of faults may differ depending on applications, most random transient faults can be corrected even with a small window size. The resulting reduction in effective fault probability can affect positively on event detection performance.

Table 1 shows how erroneous readings due to some transient faults are corrected when *M* = 4 and *δ* = 0.75. For *i* = 1, the filter at node *v*_1_ will generate 0's even if $x_{1}^{4}$ and $x_{1}^{6}$ are 1. In the case of *i* = 5, where an event occurs at time 1 and *v*_5_ is assumed to be in the event region, the output becomes 1 with a delay of two cycles. That is, $y_{5}^{3}$ becomes 1.

**Figure 2.**
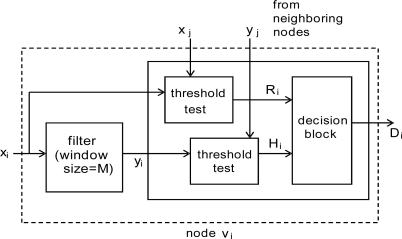
Proposed event detection scheme.

Both *x′_j_s* and *y′_j_s* will be used in event detection as shown in Figure 2, where two identical blocks are employed to perform threshold tests (to be addressed shortly) with *x′_j_s* and *y′_j_s*, respectively. The resulting binary decisions, *R_i_* and *H_i_*, will be given to the subsequent decision block to make a final decision *D_i_* on an event.

In the majority voting in [2], only the upper left threshold test block is employed like most other schemes, although the block could be functionally different. In our proposed event detection scheme both *R_i_* and *H_i_* are used. The final decision *D_i_* on an event will be made based on *H_i_*, while *R_i_* is used as a warning of an event.

### 3.3. Dynamic Threshold Selection

In this subsection, we present our adaptive event detection scheme, focusing on the threshold test block in Figure 2, where the confidence levels introduced in the previous subsection will be used to dynamically adjust the threshold for event detection. The confidence levels, updated each time event detection/fault detection is performed, are utilized to isolate potentially faulty sensor nodes and reinstate them if some given conditions are met. The resulting changes are to be reflected in the number of neighboring nodes (*i.e.*, the effective node degree $d_{i}^{k}$ at time *k*) of each node *v_i_*, and it will in turn modify the threshold *θ* for the next event detection cycle. In order to realize this adaptivity, each sensor node *v_i_* holds its fault status *F_i_*, its self-confidence level *c_i_*, the confidence levels of its neighboring nodes *w_ij_*, and the fault status of node *v_j_* from the viewpoint of *v_i_*, *F_ij_*.

The proposed event detection scheme, where the threshold *θ* is dynamically adjusted depending on the effective node degree, can be depicted as follows. Majority voting is used in the threshold test. *F_i_* and *F_ij_* are initialized to 0 (good).

**Table:** 

Adaptive Event Detection Scheme Obtain sensor reading *x_i_* and filter it to get *y_i_* Obtain sensor readings *x_j_*, filtered outputs *y_j_*, and *F_j_* from neighbors Set the threshold θ to *d_i_*/2 Determine *b_i_*, the number of neighbors with *x_j_* = *x_i_* Determine *q_i_*, the number of neighbors with *y_j_* = *y_i_* If *q_i_* ≥ *θ*, then *H_i_* ← *y_i_*, else *H_i_* ← ¬*y_i_* If *b_i_* ≥ θ, then *R_i_* ← *x_i_*, else *R_i_* ← ¬*x_i_* Report an event (*i.e.*, *D_i_* = 1) if *H_i_*=1 Report a warning if *R_i_* = 1 Update the confidence levels *c_i_* and *w_ij_*

In steps 1 and 2, each sensor node receives its own and neighbors’ sensor readings (including filtered ones). Steps 3 to 5 are functions to be performed in the two threshold test blocks in Figure 2. In step 3, the threshold value for majority voting to be used in step 5 is determined. Step 5 will set *R_i_* (*H_i_*) to either 0 or 1 depending on the number of matching neighbors obtained in step 4. *R_i_* and *H_i_* at node *v_i_* can be set against its own readings if the node fails to pass the threshold. In step 6, the decision on an event will be made. *R_i_* = 1 will be taken as a warning since it might occur due to transient faults. If it is an indication of an event, the decision on an event will be made at the time *H_i_* becomes 1. The warning must be given to its neighboring nodes to shorten the cycle time momentarily so that an event can be reported quickly. Confidence levels are updated in step 7. The confidence level of *v_j_* from the viewpoint of *v_i_*, *w_ij_*, is updated according to Table 2.

As shown in Table 2, *w_ij_* is increased by *α* only when *F_j_* = 0 (good) and *D_i_* = *y_j_*. In other words, confidence level of *v_j_* from the viewpoint of *v_i_* becomes higher when both *v_i_* and *v_j_* have similar sensor readings and *v_j_* is currently in the good state. The second and fourth rows decrease *w_ij_* by *α* since *F_j_* = 1 (faulty).

The third row can be explained using the following three representative cases among others. It lowers the confidence level of its neighboring node *v_j_* only when *D_i_* is equal to 0.

**Figure 3.**
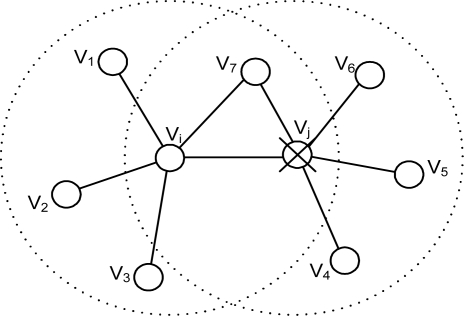
Case 1 for the third row in Table 2.

**Figure 4.**
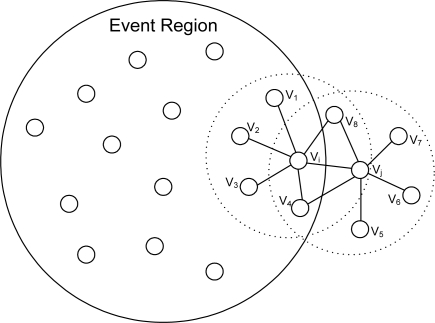
Case 2 for the third row in Table 2.

**Figure 5.**
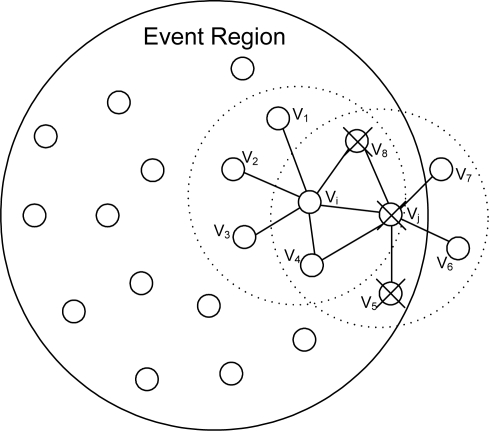
Case 3 for the third row in Table 2.

Case 1: Suppose that two good nodes *v_i_* and *v_j_* are neighboring each other and each of them is surrounded by sufficient number of good nodes to pass the threshold test. The first case occurs when *v_j_* becomes faulty and sends a 1 as shown in Figure 3. In this case, *v_i_* will have *D_i_* = 0, *y_j_* = 1, and *F_j_* = 0 (until *v_j_* sets *F_j_* to 1). Hence the conditions are met. The desired action at node *v_i_*, as far as confidence level is concerned, is to lower the confidence level of *v_j_* (*i.e.*, *w_ij_*).
Case 2: The conditions can also be met when two good nodes, *v_i_* and *v_j_*, neighboring each other are located in such a way that only one of them is in the event region, as illustrated in Figure 4. In the figure, *v_i_* is in the event region and receives a 1 from *v*_1_ through *v*_4_ and will eventually report an event (*i.e.*, *D_i_* = 1). Meanwhile, *v_j_* also makes the right decision of no-event (*i.e.*, *D_j_* = 0). When *y_i_* = 1 and *y_j_* = 0, as expected, *v_i_* will have *D_i_* = 1, *y_j_* = 0, and *F_j_* = 0, satisfying the conditions. The conditions are also met for *v_j_* since *D_j_* = 0, *y_i_* = 1, and *F_i_* = 0. The correct action in case 2, as far as confidence level is concerned, is as follows: (a) at node *v_i_*, *w_ij_* needs to be increased, (b) at node *v_j_*, *w_ji_* also needs to be increased.
Case 3: It occurs when faulty nodes in close proximity, claiming to be good, are in an event region as shown in Figure 5 such that their readings are 0 as opposed to 1 (abnormal). Suppose that two nodes in the event region, *v_i_* and *v_j_*, are neighboring each other and *v_j_* is one of the faulty nodes. Apparently *v_j_* may have *D_j_* = 0 since *v*_6_ and *v*_7_ are likely to report a 0 since they are outside the event region. Both *v_i_* and *v_j_* meet the conditions. The proper actions in this case are (a) at node *v_i_*, where *D_i_* = 1, *y_j_* = 0, and *F_j_* = 0, *w_ij_* has to be lowered, (b) at node *v_j_*, where *D_j_* = 0, *y_i_* = 1, and *F_i_* = 0, *w_ji_* needs to be increased to eventually change *F_j_* to 1.

For node *v_i_* the above cases can be divided into two groups, depending on the value of *D_i_*. The first group (*D_i_* = 0) includes case 1, case 2(b), and case 3(b). Although the three cases in the first group cannot be distinguished based on the given information, the desired actions may differ. Only case 1 wants to lower the confidence level. The second group (*D_i_* = 1) includes case 2(a) and case 3(a), requesting conflicting actions. The third row in the table allows only case 1 to update the confidence level, ignoring all other cases. The reasons for taking this action are as follows. Confidence levels are maintained to isolated nodes with permanent faults or nodes behaving incorrectly for some extended period of time. Hence it is primarily intended to handle case 1. All other cases are related to events, which in general consume a relatively small portion of the entire monitoring time. In the case of an event, due to the conflicting requests, correctly updating confidence levels needs some additional information on the exact boundary of the event region, requiring more sophisticated computations. Hence momentarily stopping the updates in the case of an event may be appropriate since the network continues its monitoring function with most of the faulty nodes isolated.

Based on Table 2 the confidence level *w_ij_* is updated as follows.

(2) $$w_{\mathrm{ij}}=\{\begin{array}{ll} \text{max}(w_{\min},w_{\mathrm{ij}}-\alpha ) & \text{if}\;(D_{i}\neq y_{j}\;\text{and}\;D_{i}=0)\;\text{or}\;F_{j}=1\\ \text{min}(w_{\max},w_{\mathrm{ij}}+\alpha ) & \text{if}\;D_{i}=y_{j}\;\text{and}\;F_{j}=0\\ w_{\mathrm{ij}} & \text{otherwise} \end{array}$$

It is increased or decreased by *α* each time the conditions are met. The value of *α* needs to be chosen depending on the types of faults and applications. If *α* is relatively small, a node with transient faults is highly unlikely to be removed from the neighbor list. As *α* increases, however, it can be removed with an increased probability. Even if it is isolated, the node with only transient faults will be reinstated in our adaptive scheme.

A potentially faulty neighboring node *v_j_* of node *v_i_* will be removed from the effective neighbor list of *v_i_* as follows. If *F_ij_* = 0 (good) and *w_ij_* = *w_min_*, *F_ij_* is set to 1 (faulty) and *v_j_* is removed from *v_i_*’s effective neighbor list. On the other hand, if *F_ij_* = 1 (faulty) and *w_ij_* = *w_max_*, *F_ij_* will be set to 0 (good) and *v_j_* will rejoin the *v_i_*’s effective neighbor list. Once a node is removed from the list (*i.e.*, *w_ij_* = *w_min_*), it can rejoin the list only when *w_ij_* is increased and reaches *w_max_*. Similarly, once a removed node rejoins the effective neighbor list, it will remain there unless *w_ij_* reaches *w_min_* again.

Similarly the self-confidence level of *v_i_*, *c_i_*, is also updated in step 7. It is lowered if the decision made at *v_i_*, *D_i_*, is different from its own sensor reading filtered, *y_i_*, except for an event.

(3) $$c_{i}=\{\begin{array}{cc} \text{max}(c_{\min},c_{i}-\alpha ) & \text{if}\;D_{i}\neq y_{i},\\ \text{min}(c_{\max},c_{i}+\alpha ) & \text{otherwise} \end{array}$$

Fault status *F_i_* changes depending on the self confidence level *c_i_*. *F_i_* will be set to 1 (faulty) when *c_i_* becomes *c_min_*. Once it is set to 1, it will stay there until *c_i_* reaches *c_max_* again.

**Figure 6.**
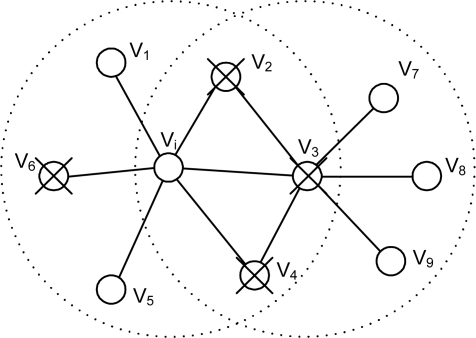
A good node failing to pass the threshold due to neighboring faulty nodes.

In the case where a good sensor node has more faulty neighbors, the node might be determined to be faulty, as illustrated in Figure 6, where *c_i_* for *v_i_* will be lowered due to the inequality *D_i_* ≠ *y_i_*. It, however, will highly likely be determined to be a good node with time. The node, *v*_3_, a neighbor of *v_i_*, will determine itself to be faulty if it cannot pass the threshold such that its confidence level *c*_3_ reaches 0. In the figure, *v*_3_ has more good neighbors than faulty ones. Hence *D*_3_ is highly unlikely to be *y*_3_. Once *F*_3_ is set to 1, *v_i_* will remove *v*_3_ from its neighbor list. As a result, its effective node degree $d_{i}^{k}$ will be lowered. If this also happens at *v*_4_, for example, the node is also removed from the list, and the node degree of *v_i_* is further lowered. Finally, *v_i_* passes the threshold, changes its fault status to 0 (good) some cycles later, and it can then be treated as a good node. If a larger number of faulty nodes are in close proximity, this recovery might not happen. The case, however, is extremely unlikely since our adaptive scheme removes faulty nodes as soon as identified. Unless all the nodes become faulty almost simultaneously, such a situation is unlikely to occur.

## 4. Simulation Results

Computer simulation is conducted to evaluate the performance of the proposed event detection scheme. Our simulated sensor network consists of 1,024 sensor nodes, randomly deployed in a 32 × 32 square region. Initially each node has about 12 neighboring nodes on average (*i.e.*, *d* ≈ 12) in the simulation. Event region is assumed to be a circle with radius *l* = 2*r*, where *r* is the transmission range of each sensor node. Nodes with a permanent fault are assumed to consistently report an unusual reading (similar to stuck-at-1) or a normal reading (similar to stuck-at-0) with the same probability of 0.5, irrespective of the regions they are in. Both permanent and transient faults are considered and their probabilities are denoted by *p_p_* and *p_t_*, respectively. Hence the overall fault probability *p* is equal to *p_p_* + *p_t_*. In filtering transient faults, *M* (window size) and *δ* (threshold) are set to 4 and 0.75, respectively. In the simulation, three different values of *α*, 0.1, 0.2 and 0.3, are chosen for comparison purposes.

Three metrics, DA(event detection accuracy), FAR (false alarm rate) and ERDR (event region detection rate), are used to evaluate the performance of the proposed event detection scheme. FAR is defined as the ratio of the number of nodes reporting an event, in the case of no event, to the total number of sensor nodes. DA is the ratio of the number of times that events are detected to the total number of event occurrences. ERDR is the ratio of the number of nodes, in the event region, reporting an event (*i.e.*, *D_i_* = 1) to the total number of nodes in the event region. Our objective is to keep high DA and low FAR simultaneously even when the fault probability is high. Although ERDR is not the main concern in this paper, statistical data for event region detection are obtained for future research.

Table 3 shows DA for the proposed event detection scheme for various values of *p_t_* when *p_p_* is increased by 0.01 every 20 cycles up to 0.5. Based on the results we can claim that DA can be maintained high even with increasing number of faults.

**Figure 7.**
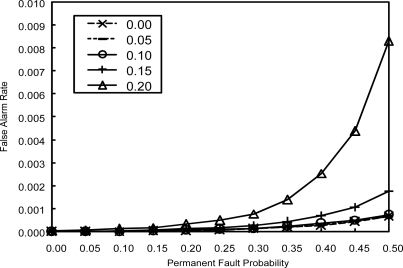
FAR with increasing *p_p_* for various values of *p_t_*.

Figure 7 shows FAR with increasing permanent fault probability *p_p_* for various values of transient fault probability *p_t_* when *α* = 0.2. To see how the proposed scheme adapts to the increase in the number of faults, *p_p_* is increased by 0.01 every 20 cycles. FAR is kept very close to zero even when *p_p_* is 0.5. In the case of *p_t_* = 0.1 and *p_p_* = 0.2, for example, FAR is about 0.00006. That is, only 0.06 nodes out of 1,024 make a false alarm even in the combined fault probability of 0.3. Sensor nodes with a permanent fault (producing erroneous data repeatedly for an extended period of time) can hardly affect the decision making process since they will be isolated from the network until they exhibit normal behavior again. In addition, the increase in transient fault probability *p_t_*, up to 0.2, does not cause any notable performance degradation due to the effective filtering of transient faults.

**Figure 8.**
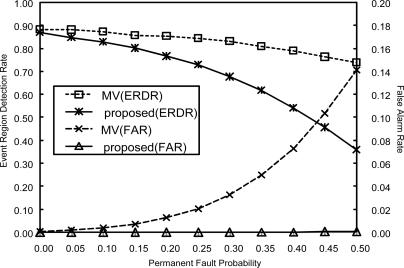
Comparison between the proposes scheme and majority voting(MV) with increasing *p_p_* when *p_t_* = 0.1.

We have compared the performance of the proposed scheme with that of the majority voting. The results for *p_t_* = 0.1, 0.0 ≤ *p_p_* ≤ 0.5, and *α* = 0.2 are shown in Figure 8. Unlike the proposed scheme, FAR for the majority voting increases with *p_p_*, exhibiting a significant amount of false alarms. These false alarms will waste the network resources, resulting in a considerable reduction in network lifetime. On the other hand, ERDR for our scheme is lower than that of the majority voting. The reason for this degradation in ERDR is that correcting erroneous readings by employing a filter may reduce the number of non-event sensor nodes incorrectly reporting a 1 (abnormal). In fact incorrect readings due to faulty sensor nodes near but outside an event region may affect positively on the event detection.

**Figure 9.**
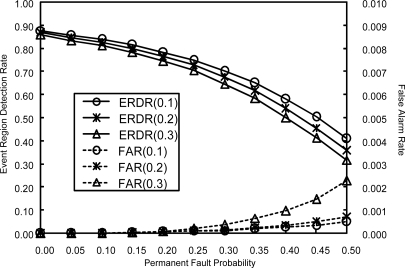
ERDR and FAR for three different values of *α* when *p_t_* = 0.1.

Similar simulation is done to compare the performance for three different values of *α*: 0.1, 0.2 and 0.3. The resulting FAR and ERDR are shown in Figure 9, where the number in the parenthesis represents the value of *α*. As can be seen, the best performance is obtained for *α* = 0.1, although the performance difference between 0.1 and 0.2 is marginal. A notable degradation in performance can be observed for *α* = 0.3. This stems from the fact that some good nodes are removed from the neighbor list due to transient faults.

**Figure 10.**
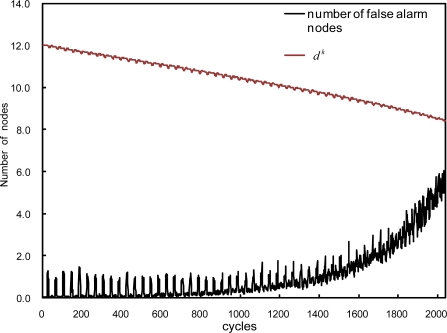
Average node degree *d^k^* and the number of false alarms when *p_p_* increases up to 0.5 and *p_t_* = 0.2.

In the proposed adaptive scheme, a sensor node *v_i_* treats a potentially faulty sensor node *v_j_* as a faulty node at the time the confidence level *w_ij_* reaches 0. The resulting reduction in effective node degree of each sensor node, $d_{i}^{k}$, will accordingly change the threshold *θ* to adapt to the new network topology. Consequently faulty nodes can only affect the decision making process until they are identified and isolated. Due to the dynamic threshold selection, high event detection performance can be maintained even with increasing fault probability as shown in Figure 10, where *p_p_* is increased by 0.01 every 40 cycles and an event is assumed to occur every 40 cycles. As expected, the average node degree *d^k^* (at time *t* = *k*) decreases and the number of false alarm nodes slowly increases with *p_p_*. The number of false alarm nodes moves up and down periodically due to the artificially generated periodic events.

**Figure 11.**
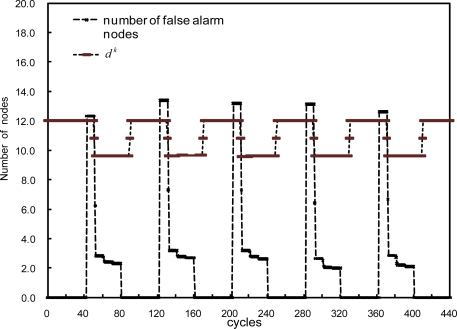
Average node degree *d^k^* and the number of false alarms when intermittent faults occur simultaneously every 80 cycles with the probability of 0.2.

Another simulation is performed to show how the proposed scheme adapts to a special type of fault, producing erroneous readings periodically for some period of time. For simplicity, each node is assumed to have such an intermittent fault with probability of 0.2 every 80 cycles, producing incorrect readings for 40 cycles. The results are shown in Figure 11, where the number of nodes that make a wrong decision soars up to more that 12 at the time such a fault occurs, but goes down to below 4 after a few threshold adjustments. Once the erroneous data due to the faults disappear, the threshold goes back to the original position, as expected.

The proposed adaptive scheme has the potential to adapt to different fault patterns. The performance of the scheme will further be investigated by generating various types of faults discussed in [12].

## 5. Conclusions

In this paper, we proposed an adaptive fault-tolerant event detection scheme for wireless sensor networks. It maintains high performance, in terms of detection accuracy and false alarm rate, for a wide range of fault probabilities, by employing a dynamically adjusted threshold and a filter for tolerating transient faults. Simulation results show that the scheme mitigates the negative influence of various types of faults by exploiting adaptation to temporal behavior of faults. Although we focused on faulty sensors, the scheme can be extended to cover faults in communication with minor modifications. Only three bits of information are exchanged each event detection cycle to reduce the communication cost. More extensive simulation is currently being conducted to estimate how the scheme performs for various event region shapes.

## Figures and Tables

**Table 1.** An illustration of filtering transient faults when *M* = 4 and *δ* = 0.75.

*i*	$x_{i}^{1}$	$x_{i}^{2}$	$x_{i}^{3}$	$x_{i}^{4}$	$x_{i}^{5}$	$x_{i}^{6}$	$y_{i}^{1}$	$y_{i}^{2}$	$y_{i}^{3}$	$y_{i}^{4}$	$y_{i}^{5}$	$y_{i}^{6}$
1	1	0	0	1	0	1	-	-	0	0	0	0
2	0	0	0	1	1	0	-	-	0	0	0	0
3	0	1	0	1	1	1	-	-	0	0	1	1
4	1	1	1	1	0	0	-	-	1	1	1	0
5	1	1	1	1	0	1	-	-	1	1	1	1

**Table 2.** Updating *w_ij_* at node *v_i_*.

*D_i_* = *y_j_*	*F_j_*	*w_ij_*
yes	0(good)	up
yes	1(faulty)	down
no	0(good)	down for *D_i_* = 0
no	1(faulty)	down

**Table 3.** DA for various values of *p_p_* and *p_t_*.

2*p_p_	*p_t_*				
	0.00	0.05	0.10	0.15	0.20

0.00	1.000	1.000	1.000	1.000	1.000
0.10	1.000	1.000	1.000	1.000	1.000
0.20	1.000	1.000	1.000	1.000	0.999
0.30	1.000	1.000	1.000	1.000	0.995
0.40	1.000	1.000	1.000	0.993	0.949
0.50	0.999	0.999	0.997	0.933	0.753
